# Anomalous values and missing data in clinical and experimental studies

**DOI:** 10.1590/1677-5449.190004

**Published:** 2019-05-21

**Authors:** Hélio Amante Miot

**Affiliations:** 1 Universidade Estadual Paulista – UNESP, Faculdade de Medicina de Botucatu, Departamento de Dermatologia e Radioterapia, Botucatu, SP, Brasil.

**Keywords:** data analysis, database, outlier, multiple imputation

## Abstract

During analysis of scientific research data, it is customary to encounter anomalous values or missing data. Anomalous values can be the result of errors of recording, typing, measurement by instruments, or may be true outliers. This review discusses concepts, examples and methods for identifying and dealing with such contingencies. In the case of missing data, techniques for imputation of the values are discussed in, order to avoid exclusion of the research subject, if it is not possible to retrieve information from registration forms or to re-address the participant.

Before embarking on the process of analyzing the data from a clinical or biomedical study, it is imperative to undertake a careful evaluation of the possibility of missing data or anomalous values in the sample, since they are commonplace and failure to detect them can compromise a study’s conclusions or its power of inference.^1^ Anomalous values can be the result of errors of recording, of typing, or of readings taken with instruments, or may be true outliers.^2^

As the sample size and/or the number of variables increase, the likelihood of input errors also increases. Studies with very large samples employ techniques such as double-input or review of sub-samples of records, to identify (and prevent) possible errors.

Table 1 shows hypothetical data from a clinical trial in which certain patterns of anomalous values, outliers, and missing data are illustrated.

**Table 1:** Example data records (hypothetical) from a clinical study.

**Identifier**	**Age** *	**Sex**	**Pregnancies**	**Systolic blood pressure** **	Diastolic blood pressure**	**Body mass index^#^**
1	46	F	2	120	80	23.8
2	50	F	3		110	24.9
3	69	F		110	150	22.9
4	22	M	0	135	85	24.1
5	555	M	0	165	95	27.0
6	38		0	125	75	23.9
7	18	F	6	155	90	26.1
9	58	F	3	135	75	24.2
10		M	0	145	85	25.8
11	93	M	0	150	115	24.1
12	45	F	1	135	135	23.7
13	43	F	1	120	80	25.1
14	38		2	140	90	25.0
15	37	M	0	235	180	29.2
16	30	M	3	130	100	24.9
17	42	N	0	120	70	23.8
18	30	F		115	75	
19	25	F	0	135	100	24.2
20	28	M	0	145	105	23.1
21	58	F	3	135	75	24.2

* Age in full years;

** Blood pressure in mmHg;

# Body mass index in kg/m^2^.

It can be observed from the sequence of participant identifier numbers that participant number 8 is not included in the records shown in Table 1, which could be because he/she was excluded from the protocol or because of a human input error.

The age column shows one participant’s age as 555 years, which is likely to be because a number key has been pressed too many times (for example, 555 instead of. 55 years). However, if the wrong number had been typed and the result is a believable value (such as 23 instead of 32 years, or 4 instead of 44 years), then visual identification of the error would be very much less likely.

Another problem related to recording participants’ age is caused by a tendency for research subjects to give their age rounded down to an age younger than their true age (for example, 40 rather than 43 years). In order to minimize this type of bias, it is recommended that participants’ year of birth, or even their full date of birth, should be recorded and then their age can be calculated later, when data analysis is conducted. In this case, care should be taken not to record the current date or year instead of the year or date of birth of the participant (for example, 2019 rather than 1979).

Participant 17’s sex was recorded as “N”, which is a code that is not used for this variable (M or F). Since “N” is the letter adjacent to “M” on the keyboard, this is also a common pattern of input error. Additionally, systems used for statistical analysis may differentiate between higher and lower case letters (for example, “M” and “m”) and may also register accents in languages that use them (for example, “não” vs. “nao” in Portuguese). These possibilities can be eliminated by using numerical codes for responses (for example, Male = 1 and Female = 2; Yes = 1 and No = 2).

Sometimes, errors can only be detected by evaluating additional variables, as is the case in record 16, where a participant listed as male reports having had three pregnancies. Along the same lines, record 7 is a participant who is only 18 years old, but reports six pregnancies. Finally, participant 21 has exactly the same records as participant 9 for all variables, suggesting double inclusion in the study.

Tests should also be conducted to detect incongruities where values have interdependent behavior. For example, diastolic blood pressure should be lower than systolic, which is not the case in records 3 and 12, in one of which there is a reversal of values and in the other the same value has been input twice.

Errors of measurement caused by incorrectly reading instruments (for example, sphygmomanometers and balances) induce a systemic error that is very unlikely to be detected and corrected. When the error is uniformly propagated throughout the sample (for example, a reading that is 10 mmHg higher for all records), it does not cause such a significant problem for internal comparison of groups. However, when different instruments with calibration problems or poor reproducibility are used, variability is increased and parameters become less exact. Precautions to ensure that data collection instruments or laboratory methods are in agreement are extremely important, because corrections for these biases made during the analytical phase (for example, transforming values into Z scores for the data collected with each instrument) have unsatisfactory performance.^3^

This is an appropriate time to mention that study participants may falsely report some types of information that involve cultural values, for reasons of acceptance, social identification, or moral judgment. In general, values reported for body weight, use of illicit substances, and number of extramarital relationships tend to be underestimated by research participants, whereas reported values for height, use of safe sex methods, and affirmative attitudes (for example, altruism, solidarity, or common sense) tend to exaggerate the true values. There is no infallible method to prevent this type of false report and neither is there any statistical method of correcting for such biases. However, in addition to using objective measures (for example, measuring weight and height during the interview, verifying year of birth on an identity document or hospital records) researchers recommend using confirmatory questions that enable the integrity of information provided to be verified (for example, at the start of the interview ask how many times per month a respondent has used illicit substances and at the end ask how many times a week they use specific substances, marijuana, cocaine, acid, etc.).

The accuracy of records is crucial for a study’s quality and the validity of its conclusions; efforts to minimize these types of problems must be considered when planning research.

Data can also contain values that are very different from the behavior of the sample. These are known as outliers and they are not recording errors, but do not fit the probability distribution of extreme values (whether higher or lower) that is found in the population. In the example shown in Table 1, participant 11 is 93 years old, and participant 15 has blood pressure that contrasts with all of the other participants’.

In normal distributions,^4^ outlier values are defined as those that are more extreme than 1.5 interquartile deviations below p25 or above p75 in a sample (Figure 1), or standardized values that are beyond three standard deviations (higher or lower) for the sample. Identification of outliers in non-normal distributions, correlation analyses, or multivariate analyses is more complex and is beyond the scope of this review.^5^^-^ 7

**Figure 1:**
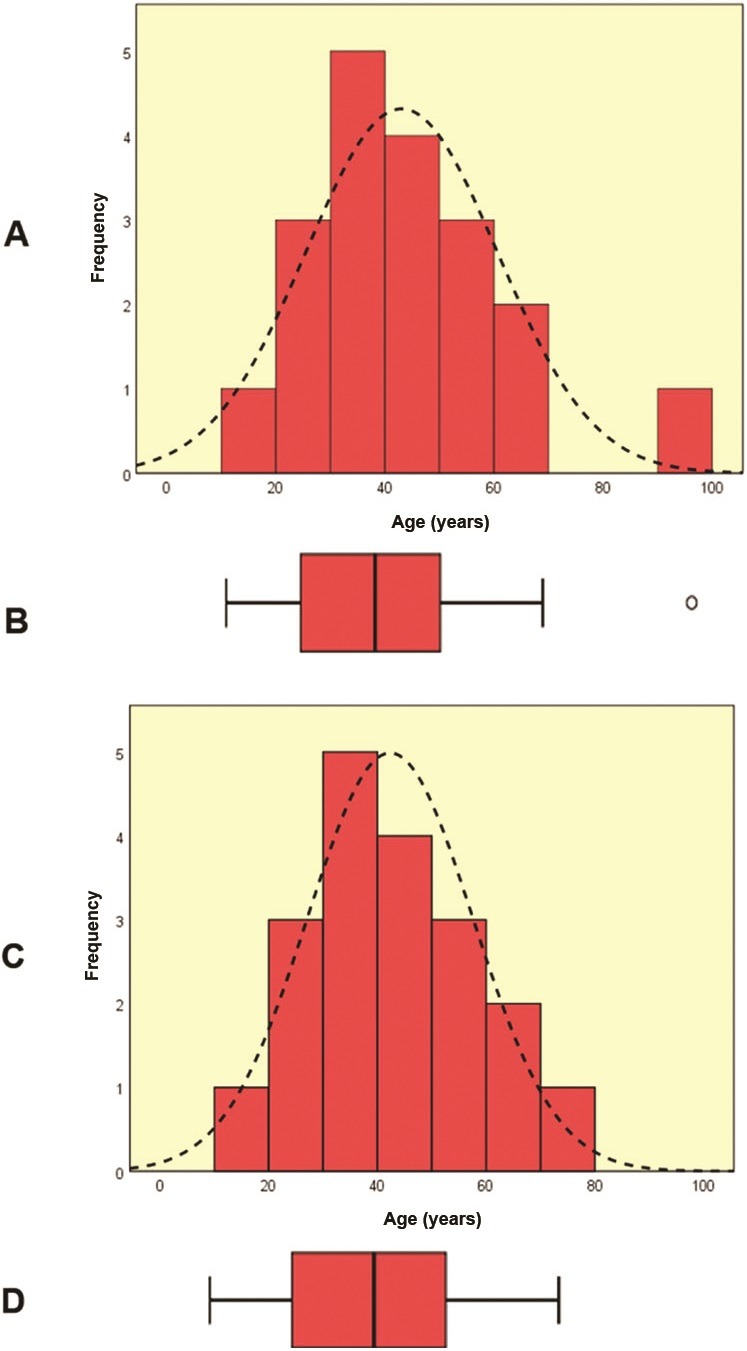
Graphs and box plots for the variable age shown in Table 1, before (A and B) abd after (C and D) winsorization. There was an outlier – the 93 years-old –, which was more extreme than the 1.5 times the interquartile deviation (25 years) added to the 75th percentile (55 years) and was Winsorized to 70 years (n = 19).

However, identification of outliers is just the first step; there is also a great matter of debate on how to deal with these data. If, on one hand, these records are out of tune with the sample, increase the variability of data, compromise the normality of the distribution, reduce statistical power, and have an impact on population inferences, on the other hand, they are real values, from subjects who were part of the study population. Outliers can even be indicative of special patterns within a sample, providing base for new hypotheses on the phenomenon studied, or may reveal underlying non-normal probability distributions in the population.^8^

The bivariate statistical tests habitually used for parametric data (Student’s *t* test, ANOVA, and Pearson’s correlation coefficients) are relatively robust to deal with a small proportion of outliers. In turn, rank-based tests (Mann-Whitney, Wilcoxon, Kruskal-Wallis, and Spearman’s coefficient) are unaffected by extreme values. The decision to exclude subjects with outlier values penalizes the sample, and should be avoided. Rather, if necessary, it is possible to deal with outliers using winsorization, or trimming, or employ clustering techniques, resampling (bootstrapping), or robust statistical analyses, which provide an approximation for a probability distribution, based on the central data.^9^^-^ 13

In winsorization, the anomalous datum is substituted with a value that is beyond that of the next nearest value, bringing the outlier closer to the remainder of the data.^1^ In the case of Table 1, the age of 93 years could be winsorized to 70 years, one unit higher than the next highest age: 69 years (Figure 1).

In trimming, a certain percentage of the extremes of the sample (for example, the most extreme 2%) is excluded bilaterally from the analysis. This procedure makes the sample more uniform, but it can be at the cost of the power of the statistical analysis, since it reduces the sample size.^1^

Clustering techniques assess patterns of proximity of participants based on the behavior of other variables, and the outlier value is substituted with the average for subjects identified as a group. Clustering techniques, imputation based on resampling methods, and robust statistical methods require the involvement of an experienced statistics professional.^10^^,^ 11 ^,^ 14 ^-^ 17

It is important that researchers employ routines for identification of anomalous values and outliers, because of the inferential cost they can impose, especially in studies with small numbers of participants. If outliers occur at low frequencies in the sample and do not change the conclusions of an analysis, it is recommended that data be not transformed in any way.

Another commonplace occurrence in clinical and experimental research is missing data, which can be easily diagnosed visually, by the “space” that they leave on a spreadsheet of data (Table 1). However, as the number of subjects and/or variables increases, it is recommended that strategies to test for missing data be adopted. Furthermore, some spreadsheet and data analysis programs automatically substitute missing data with ZERO or an incorrect value (for example, 999), which can cause even greater problems if these data are not identified.

Missing data may be caused by input errors or they may really have been unavailable when data were collected. If possible, retrieval of original records or returning to the subject for confirmation are the best solutions in these cases. In some cases, the behavior of other variables makes it possible to deduce the missing value with certainty. In Table 1, record 14 must be for a woman, since it shows two pregnancies.^2^

However, some data cannot be recovered *a posteriori* (for example, a patient has died or experimental mice have been euthanized), cannot be deduced, are affected by when they were collected, or are the result of complex experiments. These circumstances demand use of certain statistical techniques to deal with these limitations.^18^^-^ 20

The first step in dealing with missing data is to analyze the magnitude of the absence of values. Subjects missing more than 10% of data, or variables with more than 10% of missing values are not suitable for techniques for imputation of values, and retention of the subject or variable in the study should be questioned.

The second step is to analyze patterns in missing data, because techniques for imputation demand that the absence of data is relatively independent of other variables, since the lack of information may itself be linked to the behavior of one of the variables.

Missing data that do not follow any type of pattern of absence are known as missing completely at random (MCAR) data, such as when one sheet of a questionnaire is lost, a single blood sample coagulates, or a patient moves to another town. In such cases, it is assumed that the absences of data are caused by elements external to the protocol, and so analysis of the data with or without those participants with missing data will not change the magnitude of the effect.^21^

There are also missing at random (MAR) data, where the lack of one value is subject to the effect of a secondary covariable: those with less education may leave responses unanswered because they don’t understand, questions of a sexual nature may be ignored by promiscuous participants, or X-rays may be cancelled for obese patients because the equipment is not compatible. Here the results of analysis of the data with these participants may be different from the results if they are excluded; however, a significant change to the direction of the effect is not expected.^19^

Nevertheless, the most common pattern of missing data is directly related to the behavior of the variable being studied. For example, patients suffering little pain are more likely to conclude a questionnaire on symptoms; dropping out of a study might be more common among those who experience adverse effects or in a placebo group (less clinical effect); or even, more severe hypertensive patients may not attend visits to have blood pressure measured, because they are more likely to have to attend the emergency room or because of headaches. These data are missing not at random (MNAR), and they cause serious selection bias in a sample, compromising generalization of results.

If there is a small percentage of missing data and they have a random pattern (MAR or MCAR), there are a number of options for imputation. Data with a non-random pattern of absence (MNAR) demand for support from a statistical professional with experience in identification and treatment of these data.

Exclusion of the full record (all data) for participants that have missing data values (casewise or listwise) reduces the total sample size and can penalize the inferential power of the analysis if the sample is small, or, in cases in which the pattern of absence is non-random (MNAR), it can cause analytical bias. One option is to only exclude the subject from analyses of the missing variables (pairwise), reducing the sample size of descriptive statistics for these variables only or in analyses (for example, correlations) that employ that variable, allowing the remainder of the data available on the subject to be used in other statistical analyses.^22^

Substitution of the missing value by an estimator of the central tendency (mean, mode, or median) of the other values for the variable is a relatively precise option, but it reduces the variability of data (overfit) and does not consider the effect of other variables in imputation. On the other hand, substitution of the missing value by the value in the adjacent record (value for the previous or next subject) increases the variability of data (underfit), and also does not take other variables into account. Use of multivariate regression techniques to estimate the missing value as a function of the remaining variables offers the most precise estimation, but reduces the variability of the data (overfit). These options are most appropriate when the magnitude of missing data is small (< 5%).

The best technique for substitution of absent values is multiple imputation, which employs several predictive models to validate values by testing a selection of different missing data, in order to maintain the same variance as the available values for the variable (minimizing overfit). Multiple imputation of absent values gives better analytical performance than exclusion of cases (listwise) or variables (pairwise) with missing values. In general, the multiple imputation model should contain all of the study variables, and at least 10 attempts (iterations) should be run to arrive at the best estimation of the missing data.^23^^-^ 29

Returning to the example in Table 1, the correlation between values for systolic blood pressure and body mass index is ρ = 0.60 (p = 0.01) for the 17 original pairs of data, and ρ = 0.61 (p < 0.01) after multiple imputation of the two missing values.^30^ These values show that multiple imputation techniques do not interfere with the magnitude of the effect (for example, Spearman’s ρ, odds ratios, β coefficients of regressions), but they do increase the analytical power and the precision of estimates.^21^^,^ 27

It is important to point out that these multiple imputation are not applicable to studies of just one variable, losses with a MNAR pattern, or for when the intention is to increase (artificially) the sample size. Additionally, imputation of the dependent variable (principal study outcome) on the basis of its covariates is not recommended.^29^^,^ 31

There is a special case of missing data which is the set of data that is lost because of participants who leave the study. These events are known as dropouts and they are the cause of a profusion of academic discussions on analysis of longitudinal studies (for example, cohorts and clinical trials).^32^^-^ 39 Nevertheless, as mentioned earlier, dropouts or losses to follow-up exceeding 10% of participants can seriously compromise the results of a study, except in survival studies, in which the principal outcome is itself time of survival.^40^ Dropouts can also be the result of events, which may or may not be linked to other study variables (for example, failure to attend because of an adverse event related to treatment), and analysis of the results of a study with exclusion of participants that drop out (per protocol analysis) can give a false estimate of the effect or safety of a treatment.^34^^,^ 35 ^,^ 41

Longitudinal intervention studies (for example, randomized clinical trials) should preferably analyze all participants by intention to treat (ITT), so that all of those randomized and allocated to a group should be analyzed at the end of the study, irrespective of diversions from the therapeutic protocol (for example, withdrawal or change of treatment) or of dropouts. For dropout cases, one option for ITT analysis of missing dependent variables is to copy the value from the subject’s last visit, known as last observed carried forward (LOCF), although it tends to underfit estimations of the parameter and can reduce the effect of treatment.^42^^,^ 43 Recovering the information is preferable to LOCF, even on a date long after that scheduled for the visit. Additionally, some techniques for analysis of longitudinal studies (generalized linear mixed-effects models) can deal with missing data and dropouts in their analytical structures.^35^^,^ 37 ^,^ 39 ^,^ 44 ^-^ 48

In general, descriptive statistics and bivariate analyses should be conducted including the outlier values (untransformed) and should also consider missing data, to preserve the fidelity of the description of the original sample. The techniques described here are preferred to ensure successful multivariate analyses, where the existence of outlier values or missing data can violate the preconditions of the statistical tests (for example, normality) or require exclusion of subjects and variables from the study.

Finally, the strategies used to deal with missing data and outliers should be described in detail in the methodology and when presenting the results. Irrespective, it is a good practice to conduct an analysis of the sensitivity of the results, running the same data analyses with the original values and after exclusion of cases with missing data and outliers, to test whether the direction of the results is aligned with the conclusions reached at using corrected data.^21^^,^ 36 ^,^ 49 ^,^ 50
