# Supplementary material for: Anomalous values and missing data in clinical and experimental studies
Source: J Vasc Bras. 2019 May 21;18:e20190004. doi: 10.1590/1677-5449.190004 (PMC6634950; doi:10.1590/1677-5449.190004)
Supplement: Translation

# Translation: Valores anômalos e dados faltantes em estudos clínicos e experimentais

**Authors:** Hélio Amante Miot

**Affiliations:** 1 Universidade Estadual Paulista – UNESP, Faculdade de Medicina de Botucatu, Departamento de Dermatologia e Radioterapia, Botucatu, SP, Brasil.

**Keywords:** análise de dados, base de dados, discrepância, imputação múltipla

## Abstract

Durante a análise dos dados de uma pesquisa científica, é habitual deparar-se com valores anômalos ou dados faltantes. Valores anômalos podem ser resultado de erros de registro, de digitação, de aferição instrumental, ou configurarem verdadeiros *outliers.* Nesta revisão, são discutidos conceitos, exemplos e formas de identificar e de lidar com tais contingências. No caso de dados faltantes, discutem-se técnicas de imputação dos valores para evitar a exclusão do sujeito da pesquisa, caso não seja possível recuperar a informação das fichas de registro ou reabordar o participante.

Antes de iniciar o processo de análise dos dados de uma pesquisa clínica ou biomédica, é imperioso avaliar cuidadosamente a existência de dados faltantes ou valores anômalos na amostra, pois eles são habituais, e a sua inobservância pode comprometer as conclusões do estudo ou seu poder inferencial^1^. Valores anômalos podem ser resultado de erros de registro, de digitação, de aferição instrumental ou configurarem verdadeiros *outliers* 2.

À medida que a amostra e/ou o número de variáveis aumenta, cresce a chance de ocorrerem erros de digitação. Em estudos com amostras vultosas, utilizam-se inclusive artifícios como dupla digitação ou revisão amostral dos registros, para identificar (e prevenir) possíveis erros.

A Tabela 1 apresenta dados hipotéticos de uma pesquisa clínica em que ocorrem alguns padrões de valores anômalos, *outliers* e dados faltantes.

**Tabela 1:** Exemplo de registro de dados (hipotéticos) de uma pesquisa clínica.

**Identificador**	**Idade** *	**Sexo**	**Gestações**	**Pressão arterial sistólica** **	Pressão arterial diastólica**	**Índice de massa corporal^#^**
1	46	F	2	120	80	23,8
2	50	F	3		110	24,9
3	69	F		110	150	22,9
4	22	M	0	135	85	24,1
5	555	M	0	165	95	27,0
6	38		0	125	75	23,9
7	18	F	6	155	90	26,1
9	58	F	3	135	75	24,2
10		M	0	145	85	25,8
11	93	M	0	150	115	24,1
12	45	F	1	135	135	23,7
13	43	F	1	120	80	25,1
14	38		2	140	90	25,0
15	37	M	0	235	180	29,2
16	30	M	3	130	100	24,9
17	42	N	0	120	70	23,8
18	30	F		115	75	
19	25	F	0	135	100	24,2
20	28	M	0	145	105	23,1
21	58	F	3	135	75	24,2

* Idade em anos completos;

** Pressão arterial em mmHg;

# Índice de massa corporal em kg/m^2^.

A observação da sequência dos identificadores evidencia que o participante número 8 não foi incluído nos registros da Tabela 1, o que pode decorrer da exclusão protocolar ou de falha do digitador.

Na coluna da idade, há um participante com 555 anos, uma provável digitação múltipla de um algarismo (por exemplo, 55 *versus* 555 anos). Todavia, caso ocorresse troca de algarismos que resultasse em um valor coerente (como 23 *versus* 32 anos, ou 4 *versus* 44 anos), a identificação visual do erro seria muito dificultada.

Ainda sobre o registro da idade, há tendência dos sujeitos da pesquisa referirem sua idade arredondada para um valor abaixo do real (por exemplo, 40 em vez de 43 anos). A fim de minimizar esse tipo de viés, recomenda-se o registro do ano de nascimento, ou mesmo a data completa, sendo a idade calculada posteriormente na fase de análise dos dados. Nesse caso, deve-se ter atenção de não registrar a data ou ano atuais em vez da data ou ano de nascimento do participante (por exemplo, 2019 em vez de 1979).

O sexo do participante 17 foi registrado como “N”, uma codificação não utilizada para essa variável (M ou F). Visto que “N” é uma letra adjacente ao “M” no teclado, trata-se também de um padrão habitual de falha na digitação. Além disso, os sistemas usados para análise estatística podem diferenciar maiúsculas de minúsculas (por exemplo, “M” de “m”) e também a acentuação (por exemplo, “não” e “nao”). Tais contingências são prevenidas pela codificação numérica das respostas (por exemplo, Masculino = 1 e Feminino = 2; Sim = 1 e Não = 2).

Eventualmente, a percepção do erro depende da avaliação de mais variáveis, como ocorre no registro 16, em que um participante do sexo masculino refere três gestações. Da mesma forma, no registro 7, uma participante de apenas 18 anos refere seis gestações. Por fim, o participante 21 apresenta exatamente os mesmos registros que o participante 9 em todas as variáveis, sugerindo dupla inclusão no estudo.

Incongruências também devem ser verificadas diante de valores que apresentem comportamentos dependentes. No caso, a pressão arterial diastólica deve ser menor que a sistólica, o que não se observa nos registros 3 e 12, nos quais ocorreu inversão dos registros e duplicação do valor digitado, respectivamente.

Erros de aferição de diferentes instrumentos (por exemplo, esfigmomanômetros e balanças) induzem ao erro sistemático, muito difícil de ser identificado e corrigido. Quando tal erro se propaga homogeneamente na amostra (por exemplo, aferição de 10 mmHg a mais para todos os registros), não acarreta grande prejuízo na comparação interna dos grupos. Entretanto, quando se usam diferentes instrumentos com problemas de calibragem ou baixa reprodutibilidade, há aumento na variabilidade e perda da exatidão dos parâmetros. É fundamental a preocupação com a concordância dos instrumentos de coleta dos dados ou dos métodos laboratoriais, pois a correção desses vieses na fase analítica (por exemplo, transformação em valores de Z-score para os dados de cada instrumento) tem performance insatisfatória^3^.

Nesse ínterim, é conveniente comentar que algumas informações imbuídas de algum valor cultural podem ser falsamente reportadas pelos participantes, com a finalidade de aceitação, identificação social ou de julgamento moral. De uma maneira geral, peso corporal, uso de substâncias ilícitas e número de relacionamentos extraconjugais tendem a ser subestimados pelos sujeitos da pesquisa, enquanto a estatura, prática de sexo seguro e atitudes afirmativas (por exemplo, altruísmo, solidariedade ou bom senso) são reportados acima dos reais valores. Não há uma forma infalível de prevenir esse tipo de falso relato, tampouco a estatística pode corrigir tais vieses. Entretanto, os pesquisadores recomendam, além de medidas objetivas (por exemplo, medir o peso e a altura durante a entrevista, verificar o ano de nascimento em documento ou registro hospitalar), o uso de questões/perguntas confirmatórias que permitam verificar a integridade da informação (por exemplo, no início da pesquisa questionar o número de vezes por mês que usa substância ilícita e, ao final da entrevista, questionar o número de vezes por semana que usa substâncias, discriminando maconha, cocaína, ácido, etc.).

A exatidão dos registros é primordial para a qualidade do estudo e a validade das suas conclusões; dessa forma, o planejamento da pesquisa deve considerar esforços para minimizar esse tipo de situação.

Existem, ainda, valores que se distanciam muito do comportamento da amostra, os chamados *outliers*, que não se referem a erros de registro mas sim ao encontro probabilístico de valores extremos (para mais ou para menos) que realmente existem na população. No exemplo da Tabela 1, o participante 11 tem 93 anos, e o participante 15 apresenta níveis pressóricos discrepantes dos demais.

Em distribuições normais^4^, valores *outliers* são definidos como os mais extremos que 1,5 desvio interquartílico abaixo do p25 ou acima do p75 de uma amostra (Figura 1), ou valores padronizados que ultrapassem três desvios padrão (para mais ou para menos) na amostra. Em distribuições não normais, análises de correlações ou análises multivariadas, a identificação de valores *outliers* é mais complexa, e ultrapassa o escopo desta revisão^5^^-^ 7.

**Figura 1:** Histograma e diagrama *box plot* da variável idade apresentada na Tabela 1 (A e B) e após sua winsorização (C e D). Houve um valor *outlier* – a idade de 93 anos –, situado mais extremamente que 1,5 vezes o desvio interquartílico (25 anos) somado ao percentil 75 (55 anos), que foi winsorizado para 70 anos (n = 19).

Além da identificação de *outliers*, há grande discussão sobre como lidar com esses dados. Se, por um lado, esses registros destoam da amostra, aumentam a variabilidade dos dados, comprometem a normalidade da distribuição, reduzem o poder estatístico e influenciam na inferência populacional, por outro lado, são valores reais, de sujeitos que participam da população do estudo. *Outliers* podem, inclusive, ser indicadores de padrões especiais dentro da amostra, fundamentando novas hipóteses para o fenômeno estudado, ou ainda revelar distribuições de probabilidade não normais subjacentes da população^8^.

Os testes estatísticos bivariados habituais para dados paramétricos (teste *t* de Student, ANOVA, coeficiente de correlação de Pearson) são relativamente robustos para lidar com uma pequena frequência de *outliers*. Já os testes baseados em postos (Mann-Whitney, Wilcoxon, Kruskal-Wallis, coeficiente de Spearman) não são afetados por valores extremos. Dessa forma, a decisão de excluir sujeitos com valores *outliers* penaliza a amostra, e deve ser evitada. Em vez disso, se necessário, é possível tratar *outliers* com winsorização ou *trimming*, ou empregar técnicas de agrupamento (clusterização), reamostragem (*bootstrap*) ou análises estatísticas robustas, que promovem uma aproximação para uma distribuição de probabilidade, baseada nos dados centrais^9^^-^ 13.

Na winsorização, o dado aberrante é substituído por um valor que supere o seu antecessor, tornando o *outlier* mais próximo do conjunto de dados^1^. No caso da Tabela 1, a idade de 93 anos poderia ser winsorizada para 70 anos, uma unidade acima do penúltimo valor mais alto: 69 anos (Figura 1).

No *trimming*, uma porcentagem dos extremos da amostra (por exemplo, os 2% mais extremos) é excluída bilateralmente da análise. Esse procedimento homogeneíza a amostra, mas pode penalizar o poder da análise estatística, por reduzir o tamanho amostral^1^.

As técnicas de agrupamento avaliam padrões de proximidade dos participantes baseados no comportamento das demais variáveis, e o valor *outlier* é substituído pela média verificada entre os sujeitos identificados como um grupo. Tanto as técnicas de clusterização e imputação baseada em reamostragem quanto os métodos estatísticos robustos exigem o envolvimento de um profissional estatístico experiente^10^^,^ 11 ^,^ 14 ^-^ 17.

É importante que os pesquisadores adotem rotinas de identificação de valores anômalos e *outliers*, devido ao ônus inferencial que eles infligem, especialmente em estudos com pequeno número de participantes. Caso *outliers* ocorram em baixa frequência na amostra e não modifiquem a conclusão da análise, recomenda-se não promover nenhuma transformação dos dados.

Outra situação habitual à pesquisa clínica e experimental são dos dados faltantes, que são facilmente diagnosticados visualmente, pelo “vazio” que infligem à planilha de dados (Tabela 1). Contudo, à medida que o número de sujeitos e/ou de variáveis aumenta, recomenda-se também utilizar estratégias de verificação da sua ocorrência. Ademais, algumas planilhas e softwares de análise substituem automaticamente os dados faltantes por ZERO ou por um valor aberrante (por exemplo, 999), o que pode acarretar ainda mais prejuízo caso esses dados não sejam identificados.

Os dados faltantes podem se originar tanto de erros de digitação quanto da sua real indisponibilidade durante a coleta. Caso se possa recuperar as fichas de registro ou reabordar o sujeito da pesquisa, essas são as melhores alternativas para essa contingência. Em alguns casos, o comportamento de outras variáveis permite deduzir com certeza o dado faltante. Na Tabela 1, o registro 14 deve se tratar de uma mulher, já que refere duas gestações^2^.

Entretanto, alguns dados não podem ser recuperados *a posteriori* (por exemplo, paciente faleceu, camundongos foram sacrificados), não podem ser deduzidos, sofrem mudança com o momento da coleta, ou resultam de experimentos mais complexos. Essas circunstâncias demandam o uso de certas técnicas estatísticas para lidar com essas limitações^18^^-^ 20.

O primeiro passo para tratar os dados faltantes é a análise da magnitude da ausência dos valores. Sujeitos com mais de 10% dos dados faltantes, ou variáveis com mais de 10% dos valores faltantes, são situações desfavoráveis ao uso de técnicas de imputação de valores, e a permanência desse sujeito ou dessa variável no estudo deve ser questionada.

O segundo passo é a avaliação do padrão de dados faltantes, já que as técnicas de imputação exigem que ausência de dados tenha uma certa independência frente às variáveis subjacentes, pois a própria falta da informação pode estar ligada ao comportamento de alguma variável.

Dados faltantes que não seguem um padrão de ausência são chamados de valores faltantes completamente aleatórios (*missing completely at random*, MCAR), como quando uma folha do questionário é perdida, uma amostra de sangue coagula, ou um paciente se muda de cidade. Nesse caso, assume-se que as ausências de respostas decorram de elementos externos ao protocolo, e que a análise dos dados com ou sem os participantes com dados faltantes não muda a dimensão do efeito^21^.

Existem também os dados faltantes aleatórios (*missing at random*, MAR), em que a falta de um valor decorre do efeito de outra covariável secundária: os menos instruídos podem deixar itens sem resposta por baixa compreensão, questões de ordem sexual podem ser negligenciadas por participantes promíscuos, ou exames radiológicos podem ser cancelados em pacientes obesos por incompatibilidade do aparelho. Aqui, a análise dos dados com os participantes pode ser algo divergente dos resultados após a exclusão desses sujeitos; entretanto, não se espera uma modificação importante na direção do efeito^19^.

Contudo, o padrão mais habitual de dados faltantes é diretamente relacionado ao próprio comportamento da variável estudada. Por exemplo, a conclusão de um questionário de sintomas é mais provável em pacientes com pouca dor; o abandono do estudo é mais comum em quem tem mais efeitos adversos ou no grupo placebo (menor efeito clínico); ou ainda, as visitas para aferição de pressão arterial podem ser perdidas entre os hipertensos mais graves, devido à maior necessidade de visitas ao pronto-socorro ou à ocorrência de cefaleia. Trata-se da perda não aleatória (*missing not -at random*, MNAR), e inflige importante viés de seleção na amostra, comprometendo a generalização dos resultados.

Caso haja uma pequena porcentagem de dados faltantes e eles apresentem um padrão aleatório (MAR ou MCAR), há diferentes opções de imputação descritas. Dados com padrão de ausência não aleatório (MNAR) demandam suporte de um profissional estatístico experiente na identificação e no tratamento dos dados.

A exclusão do registro completo (todos os dados) do participante que possua algum dado faltante (*casewise* ou *listwise*) reduz a amostra total e pode penitenciar o poder inferencial da análise quando a amostra for pequena, ou, em casos que o padrão de ausência seja não aleatório (MNAR), pode incluir viés analítico. Há, contudo, a opção de se excluir o sujeito da análise exclusiva das variáveis ausentes (*pairwise*), reduzindo o tamanho amostral apenas na estatística descritiva dessas variáveis ou em análises (por exemplo, correlações) que utilizem aquela variável, o que leva ao aproveitamento dos demais dados completos do sujeito para as demais técnicas estatísticas^22^.

A substituição do valor faltante por um estimador de tendência central (média, moda ou mediana) dos demais valores da variável é uma alternativa relativamente precisa, mas reduz a variabilidade dos dados (*overfit*) e não considera o efeito das demais variáveis na imputação. Por outro lado, a substituição do valor faltante pelo dado registrado adjacente (valor do sujeito anterior ou posterior) promove aumento da variabilidade dos dados (*underfit*), e também não pondera as demais variáveis. O uso de alguma técnica de regressão múltipla para estimar o valor faltante em função das demais variáveis apresenta melhor precisão da estimativa, mas reduz a variabilidade dos dados (*overfit*). Essas alternativas são mais indicadas quando a ausência de dados é de pequena magnitude (< 5%).

A técnica mais indicada na substituição de valores ausentes chama-se imputação múltipla, que utiliza diferentes modelos preditivos para validar os valores a partir da testagem de diferentes dados faltantes, a fim de manter a mesma variância dos valores na variável (minimiza o *overfit*). Imputação múltipla de valores ausentes resulta em melhor performance analítica que a exclusão dos casos (*listwise*) ou das variáveis faltantes (*pairwise*). Em geral, deve-se incluir no modelo de imputação múltipla todas as variáveis do estudo, e devem ser realizadas até 10 tentativas (iterações) para a melhor estimativa dos dados faltantes^23^^-^ 29.

No exemplo da Tabela 1, a correlação entre os valores de pressão arterial sistólica e índice de massa corporal é ρ = 0,60 (p = 0,01) para os 17 pares de dados originais, e ρ = 0,61 (p < 0,01) após imputação múltipla dos dois valores faltantes^30^. Esses valores evidenciam que técnicas de imputação múltipla não interferem com a dimensão do efeito (por exemplo, ρ de Spearman, *odds ratio*, coeficiente β da regressão), apenas aumentam o poder da análise e a precisão das estimativas^21^^,^ 27.

É importante salientar que essas técnicas de imputação múltipla não se aplicam a estudos com apenas uma variável, perdas com padrão MNAR, ou quando se tem intenção de ampliar (artificialmente) o tamanho de amostra. Da mesma forma, a imputação da variável dependente (desfecho principal do estudo) em função das demais covariáveis não é recomendada^29^^,^ 31.

Uma situação especial se refere ao conjunto de dados faltantes decorrentes de abandono de seguimento do estudo. Esses eventos são chamados *dropouts*, e dão origem a uma rica discussão acadêmica sobre a análise de estudos longitudinais (por exemplo, coorte e ensaios clínicos)^32^^-^ 39. Da mesma forma, como discutido anteriormente, abandonos ou perdas no seguimento de mais de 10% dos participantes podem comprometer seriamente os resultados do estudo, exceto em ensaios de sobrevivência, em que o desfecho principal é, propriamente, o tempo de sobrevida^40^. *Dropouts* também podem ocorrer devido a eventos ligados ou não às demais variáveis do estudo (por exemplo, falta na visita do estudo por um evento adverso do tratamento), e a análise dos resultados de um estudo com a exclusão dos participantes *dropouts* (*per protocol analysis*) pode promover uma falsa estimativa do efeito ou da segurança do tratamento^34^^,^ 35 ^,^ 41.

Estudos longitudinais de intervenção (por exemplo, ensaios clínicos randomizados) devem, preferencialmente, ter seus participantes analisados por intenção de tratamento (*intention to treat*, ITT), em que todos os randomizados e alocados em um grupo devem ser analisados ao final do estudo, independentemente de desvios do protocolo terapêutico (por exemplo, descontinuidade ou troca do tratamento) ou de *dropouts*. Em casos de *dropouts*, uma alternativa para a análise ITT de variáveis dependentes faltantes é replicar o valor da última visita do sujeito (*last observed carried forward,* LOCF), o que promove *underfit* na estimativa do parâmetro e pode reduzir o efeito do tratamento^42^^,^ 43. A recuperação da informação, mesmo distante da data prevista da visita, é preferível ao LOCF. Além disso, algumas técnicas de análise de estudos longitudinais (modelos lineares generalizados de efeitos mistos) lidam com dados faltantes e *dropouts* nas suas estruturas analíticas^35^^,^ 37 ^,^ 39 ^,^ 44 ^-^ 48.

De forma geral, a estatística descritiva e as análises bivariadas devem ser conduzidas com os valores *outliers* (não transformados) e considerar os dados faltantes, para se manter a fidedignidade com a descrição da amostra original. As técnicas aqui descritas são preferenciais para o sucesso das análises multivariadas, em que a existência de valores *outliers* ou dados faltantes compromete os pré-requisitos dos testes estatísticos (por exemplo, normalidade) ou implicam na exclusão de sujeitos e de variáveis da pesquisa.

Finalmente, as estratégias de tratamento para dados faltantes e *outliers* também devem ser detalhadamente descritas na metodologia e na apresentação dos resultados. Ademais, é recomendável promover uma análise de sensibilidade dos resultados, procedendo a mesma análise dos dados considerando os valores originais e excluindo os casos com dados faltantes e *outliers*, a fim de identificar se a direção dos resultados segue a mesma das conclusões com os dados corrigidos^21^^,^ 36 ^,^ 49 ^,^ 50.
